# “I want them to live their best lives:” A qualitative exploration of owner experiences with walking their cats

**DOI:** 10.1017/awf.2025.10030

**Published:** 2025-08-08

**Authors:** Alex Elford, Andrew S Cooke, Beth A Ventura

**Affiliations:** 1 Department of Life Sciences, University of Lincoln, Joseph Banks Building, Green Lane, Lincoln LN6 7TS, UK; 2 Department of Livestock and One Health, Institute of Infection, Veterinary and Ecological Sciences, University of Liverpool, Leahurst Campus, Neston CH64 7TE, UK; 3 Department of Large Animal Clinical Sciences, College of Veterinary Medicine, Michigan State University, East Lansing, MI, USA 48824

**Keywords:** Animal welfare, enrichment, feline, interview, outdoor, perceptions, pet

## Abstract

The popularity of keeping domesticated cats (*Felis catus*) indoor-only or outdoor-indoor varies according to geographical location, and both have risks and benefits. Walking cats (e.g. on leashes) may enable mitigation of roaming risks while providing outdoor access, but the practice of walking cats appears relatively uncommon and is yet to be examined in the literature. Semi-structured online interviews (21 participants across seven countries) were conducted to explore cat walking perceptions and experiences in owners who currently practise it. Interview recordings were transcribed and analysed using reflexive thematic analysis. Five main themes were generated: (1) Benefits of walking; (2) Challenges around walking; (3) Safety for walking; (4) Cat individuality and walking; and (5) Attitudes about walking across geographic contexts. Themes highlighted that participants perceived benefits of walking for both cat and owner but faced challenges largely due to dogs and their owners in addition to judgment from others in the community. The main priorities of walking were seen to be ensuring safety and attending to the individual needs of each cat. Reactions to cat walking appeared to vary according to local norms and attitudes about cats and owner-cat relationships. The subjective nature of both the concept and practice of cat walking was also emphasised. These findings provide an initial base for what the experience of walking cats can be like and highlight that further research to directly investigate the welfare impacts of walking on cats and their owners is now needed.

## Introduction

Cats are popular pets worldwide; for example, over 26% of households in the USA and 24% of UK adults own at least one cat (American Veterinary Medical Association [AVMA] 2022; People’s Dispensary for Sick Animals [PDSA] 2024). Unlike dogs, however, there is little consensus regarding if and how cats should have regular access to the outdoors. Domesticated cats (*Felis catus*) are adaptable to indoor environments and living with humans (Jongman 2007) yet still share behavioural similarities with their wild relatives (O’Brien & Johnson 2007; Stanton *et al.* 2015), including hunting, stalking, exploration, climbing, running, and jumping (as highlighted in the Felidae ethogram; Stanton *et al.* 2015). As the field of animal welfare science has moved beyond merely protecting animals from harm or distress (Farm Animal Welfare Council [FAWC] 1993) to expand focus toward maximising the potential for positive experiences (Mellor 2016), the ability to engage in rewarding behaviours (including natural locomotion and behaviour patterns) in suitable environments is therefore a particularly relevant consideration for cats kept as pets.

Pet cats are commonly kept either as indoor-outdoor (living in a home but allowed time roaming unrestrained outdoors) or indoor-only (cats do not go outside unless into a fully enclosed space on the property, such as a fenced-in patio) (Foreman-Worsley *et al.* 2021; Hill 2024). Some sources claim that for indoor-only cats, behavioural freedoms “*can be harder for owners to meet… which can result in stress and unwanted behaviours*” (PDSA 2024). Strickler and Shull (2014) found that 61% of owners of indoor-only cats reported behaviour problems, most commonly periuria and aggression toward owners. Additional studies report behavioural issues to be more prevalent in indoor-only cats compared to those with outdoor access (Amat *et al.* 2009; Tan *et al.* 2020), including house soiling and destruction (Sandøe *et al.* 2017). Indoor-only cats typically have longer lifespans than indoor-outdoor cats (Tan *et al.* 2020) but are also at increased risk of adolescent obesity and related issues (Rowe *et al.* 2015). In contrast, an indoor-outdoor lifestyle enables autonomy over natural behaviours and reduces frustration (Tan *et al.* 2020; Foreman-Worsley *et al.* 2021) but also comes with risks, including road traffic accidents, other animals, malicious people, falls or traps, hazardous substances/items, and disease (Kasbaoui 2016; Tan *et al.* 2020). Additionally, unneutered roaming cats may add to homeless cat populations, and successful hunting behaviour significantly impacts wildlife (Hall *et al.* 2016; Tan *et al.* 2020).

Choices to keep cats indoor-outdoor or indoor-only vary geographically, with indoor-only being particularly common in North America (80.6%) compared to Australia (42.2%) and Europe (30.2%) (Foreman-Worsley *et al.* 2021). These differences may be partially explained by some risks being applicable only to certain regions; for example, North American natural predators such as coyotes (*Canis latrans*) or bears, such as the American black bear (*Ursus americanus*), are not present in the UK or parts of Europe or Australasia. Additional factors such as owner age, cat age, breed, and neighbourhood type also impact cat management choices (Foreman-Worsley *et al.* 2021). Legislation on cat roaming also varies geographically; some areas of North America, Australia and New Zealand enforce bylaws against roaming (Hill 2024), whereas the UK has no laws regarding cat containment, and roaming is more widely socially accepted. Additionally, differences in how owners conceptualise their cat (as a ‘child’, ‘teenager’ or ‘free agent’) can also impact choices in management (Hill 2024). As such, decisions to allow outdoor access for cats are mediated by a range of factors.

It is the norm in many countries for pet dogs to be exercised outdoors daily, often in the form of leashed walking or off-leash time with their owner (PDSA 2021; American Society for the Prevention of Cruelty to Animals [ASPCA] 2024). The physical and psychological benefits of walking for both dogs and their owners are well-described (Cutt *et al*. 2007; Curl *et al*. 2017; Westgarth *et al*. 2017, 2019; ASPCA 2024; PDSA 2024). In light of the lack of consensus on the degree and nature of outdoor access for cats (Foreman-Worsley *et al.* 2021; Hill 2024), walking them on leash, similarly to dogs, may be considered as an alternative or even ‘compromise’ style of management. Cat walking and ‘adventure cats’ (those who “*accompany their owners on outdoor excursions*” [Adventure Cats LLC 2024], e.g. camping, hiking, or more prolonged travel) are anecdotally growing in popularity worldwide, with many owners sharing their experiences online (McCann 2021; Adventure Cats LLC 2024; Rack 2024). Among animal welfare organisations, walking cats in this way appears to be a divisive proposition (British Columbia SPCA [BC SPCA] 2024; Cats Protection 2024; Rack 2024), perhaps in part because the practice of walking cats has received little to no attention in the scientific literature (and what does appear seems to be restricted to limited mention to ‘supervised’ outdoor time [Canadian Federation of Humane Societies [CFHS] 2017] or proposed ‘leash or harness walks’ [Tan *et al.* 2020]). Thus, without explicit or transferable definitions of the concept, foundational studies to examine the practice and its variation, and critically, examination of potential welfare impacts on cats, there is a lack of evidence from which to build recommendations.

It is within this context that we selected a qualitative semi-structured interview approach to explore what cat walking looks like for owners who currently practise it. We were specifically interested in exploring the following questions:

- How do cat-walkers perceive the experience of walking their cats (both benefits and challenges/barriers)?
- What do owners who currently walk cats prioritise and perceive to be important within this activity?
- How may locational context frame owners’ perceptions of and experiences with cat walking?

## Materials and methods

### Ontology and epistemology

The current study adopts a Critical Realist approach, acknowledging that an objective reality exists, but is observed, understood and defined by individuals through their own subjective lens, formed from experiences and internalisations (O’Mahoney & Vincent 2014). This framework finds value in both Positivist empirical analysis of phenomena and a Social Constructionist perspective in which concepts and meanings are actively constructed within human interaction and perception (Burr 2015). This Critical Realist underpinning was appropriate and necessary to explore the physical enactment of cat walking alongside the subjective experiences of participants and their cats and executed using qualitative data collection. The interview schedule created was focused on how participants understood cat walking, how they created meaning around the practice, and how this related to their individual lived experiences. In pursuing this approach, the data gathered was rich in detailing the context of each owner and cat in relation to their practices, allowing us to explore the phenomenon of walking cats.

### Ethical approval

The study received ethical approval from the University of Lincoln Research Ethics Committee (UoL2024_1069). All participants were required to be over 18 years old and provide digital consent to participation, having read a Participant Information Sheet outlining the research objectives and methods.

### Recruitment

We were interested in recruiting participants anywhere in the world who currently walk their cat, but did not define ‘walking’ during recruitment or in the interview to allow for participant interpretation. Purposive and snowball sampling were used due to the specificity of the population of interest, with the study advertisement posted in population-specific groups on Reddit and Facebook, in addition to sharing on the authors’ social media platforms. Eligibility criteria were specified as being an English speaker, over the age of 18, and owning at least one cat who was taken for walks. A target of 20 participants was set, giving a range of perspectives whilst also optimising chances for data saturation (Saunders *et al.* 2017). Participant recruitment and interviews were carried out in June 2024. Twenty-seven individuals were initially recruited; six later dropped out or ended up not attending their interview, leaving 21 people who were interviewed and whose data are reported here.

### Study design

A short survey on the GDPR-compliant JISC Online Surveys platform was first used to collect demographic information regarding participants and their cats (Tables 1 and 2). Participants were then interviewed on Microsoft Teams® by AE. Both the survey and interview schedule (see Supplementary material) were piloted with peers at the study institution and clarified prior to use with participants. The semi-structured interviews formed the main data collection, suitable for an exploratory study with a Critical Realist underpinning, utilising a schedule of questions whilst allowing for elaboration where appropriate, and yielding saturated qualitative data (determined when no new thematic content was brought up by continued interviewing) (Willig 2022). Interview topics covered the cat and owner’s general and walking history, experiences, perceptions, and practices. Interviews were organised to last around 1 h (range 27 to 67 min, with a mean of 49 min) and were recorded using the function on Microsoft Teams®. AE also took notes on the interview content directly after each interview using the technique of memoing to capture the researcher’s initial thoughts and ideas after data collection for reflexivity (Birks *et al.* 2008). In one case, the recording was corrupted; here, the memoing notes were used as the participant’s data to supplement the other 20 interviews (Tessier 2012).

**Table 1.** Demographic characteristics of cat owners (n = 21) interviewed regarding their experiences with taking their cats on walks

Demographic	n	Percentage
Gender		
*Woman*	17	81.0%
*Man*	2	9.5%
*Non-binary*	2	9.5%
Age (in years)		
*18–25*	4	19.0%
*26–35*	5	23.8%
*36–45*	3	14.3%
*46–55*	5	23.8%
*56–65*	3	14.3%
*65+*	1	4.8%
Location		
*United States*	7	33.3%
*Canada*	4	19.0%
*England*	5	23.8%
*Scotland*	2	9.5%
*The Netherlands*	1	4.8%
*France*	1	4.8%
*Australia*	1	4.8%
Accommodation type		
*House/Detached*	7	33.3%
*Duplex/Semi-Detached*	2	9.5%
*Townhome/Terraced*	4	19.0%
*Apartment/Flat*	8	38.1%
Outdoor areas available for cat’s access off-leash		
*None*	13	61.9%
*Netted Balcony*	2	9.5%
*Catio*	3	14.3%
*Garden/wider land (free-roaming)*	3	14.3%
Outdoor access type (for cats with outdoor areas)		
*N/A*	13	61.9%
*Restricted (human allows access when they deem appropriate)*	2	9.5%
*Semi-restricted (human allows whenever the cat wants)*	4	19.0%
*Unrestricted (access is constantly open)*	2	9.5%

**Table 2.** Characteristics of cats (n = 36) whose owners (n = 21) were interviewed regarding their experiences with walking

Characteristic	n	Percentage
Sex		
*Female*	16	43.2%
*Male*	21	56.8%
Age		
*0–1y*	4	10.8%
*2–4y*	18	48.6%
*5–7y*	11	29.7%
*8–11y*	3	8.1%
*Unspecified*	1	2.7%
Breed		
*Domestic mixed breed*	17	45.9%
*Ragdoll*	5	13.5%
*Persian*	5	13.5%
*Bengal*	4	10.8%
*Siamese*	3	8.1%
*Abyssinian*	1	2.7%
*Unspecified*	2	5.4%

### Data analysis

Demographic survey information was summarised descriptively. Interview recordings were transcribed *verbatim* by AE and resulted in 152 pages of text. Reflexive thematic analysis (Braun & Clarke 2019, 2021) was used to explore the qualitative data and inductively generate a thematic overview of cat walking. This process followed the ‘recursive’ six steps of analysis outlined by Braun and Clarke (2021; p 39): (1) Becoming familiar with the data through multiple reads and note-taking; (2) Generating initial codes (labels) for each section of transcription text; (3) Collating codes and grouping into themes; (4) Reviewing themes; (5) Defining and refining of themes; and (6) Selection of quote extracts and reporting. Some text was coded multiple times due to containing several thoughts. Braun and Clarke’s (2019) updated analysis procedure highlights the need for transparency and mindfulness from the researcher on their own active involvement in the analysis and their subjective lens, "*querying the assumptions we are making in interpreting and coding the data*” (Braun & Clarke 2019; p 594); codes and themes were revisited and clarified numerous times to lessen the impact of researcher subjectivity (Braun & Clarke 2021).

### Positionality

As qualitative researchers, we serve as instruments in the research process and therefore account for the ways in which our experiences and backgrounds may influence this process (Holmes 2020). AE was raised and resides in the UK, researches human-animal relationships, has an academic background in psychology and clinical animal behaviour and has previously worked with rescue animals. She lives with one indoor-outdoor cat and one indoor-only cat. BV has worked primarily in academia as an animal welfare scientist and frequently employs qualitative methods to explore the relationship between humans and animal welfare. She was based in North America for most of her training and career but has worked in the UK for three years. She lives with one beloved cat who is housed primarily indoors but is harness-trained and has on-leash supervised access to the outdoors around his home. ASC is a bioveterinary scientist from the UK. His research takes a One Health perspective and seeks to find opportunities to improve animal, environmental, and human well-being. He cares for two cats, previously indoor-outdoor, but now indoor-only. We collectively came to this work with the intent to understand the lived realities of cat owners who take their cats on walks. We acknowledge the impact of our own subjective frameworks and experiences, both in the design and analysis within the study. The research design underwent collaborative revision until precisely reflective of the study aims, and AE engaged in reflexive journal practices alongside memoing and code/theme revision whilst collecting and analysing data to ensure themes generated were reflective of the data.

## Results and Discussion

### Participant demographics

Participants ranged in age from 22 to 70 years old (mean = 42) and were predominantly women (81%). Respondents came from seven countries, spanning the regions of North America (n = 11; 52%), Europe (n = 9; 43%), and Australia (n = 1; 5%) (Table 1).

### Cat demographics

Collectively, the participants owned 36 cats (aged 10 months to 11 years, mean = 3.4 years) that they took for walks. All cats were neutered or spayed. Of the cats, four (11.1%) were walked only on the home property, 16 (44.4%) walked around residential and local areas (including in towns, parks, and nature reserves), and 16 (44.4%) were widely travelling ‘adventure cats.’ Aside from walks, 29 (80.6%) of the cats walked were kept indoor-only or only allowed into enclosed outdoor areas, whilst seven (19.4%) were also allowed to free roam. Of the 21 participants, 15 (71.4%) had multi-cat households; eight (53.3%) walked all the cats they had, and seven (46.6%) walked certain cats but not others (Table 2).

### Qualitative results and general discussion

**Figure 1.**
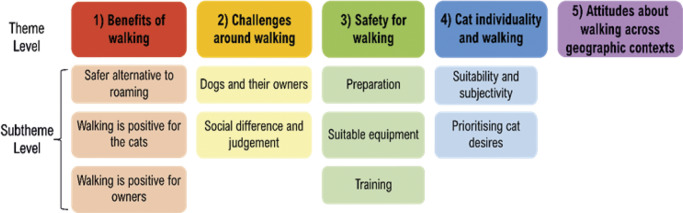
Thematic map of generated themes and subthemes summarising owners’ experiences with walking their cats.

Five main themes were generated through the analysis: (1) Benefits of walking; (2) Challenges around walking; (3) Safety for walking; (4) Cat individuality and walking; and (5) Attitudes about walking across geographic contexts. These themes and their respective subthemes are visualised in the thematic map (Figure 1). Themes are explored below, utilising quotes from participants, in which participants are represented using abbreviation IDs capturing their location (e.g. US1 is a participant from the United States of America; SCO2 is from Scotland, etc). Cats were given pseudonyms for reporting to protect participant anonymity.

### Theme 1: Benefits of walking

#### Safer alternative to roaming

Eighteen out of 21 participants kept cats indoors or with access only to enclosed outdoor spaces, aside from walking. When discussing these management choices, unsupervised roaming was generally regarded by participants as “*too dangerous*” [CAN2], with some taking a more emotive stance of it being considered as “*wildly irresponsible* … [and] *lazy ownership*” [SCO2]. Participants raised specific concerns, including predators, road traffic accidents, and the impact of cats on wildlife. For example, US1 explained how she has “*an eagle that lives … within like a mile of us, and it would definitely pick up a tiny cat like her.*” CAN3 showed similar concerns: “*coyotes have moved into Toronto just in the last ten years … to the point where you worry about them … the vets are always saying, you know, they have to stay indoors.*” Road traffic accidents were both a risk and a reality that many participants had experienced previously with roaming cats. SCO1 explained how they determined their cat ‘Batman’s’ lifestyle based on past negative experiences: “*I was like, ‘I don’t want him to be an outdoor cat’ because my first ever cat got hit by a car and I’d only had him for five months.*” The risks of roaming to wildlife were also important to participants, as US5 explained: “*I’m in a number of, like, native plant groups and ecological awareness stuff…there’s always a lot of conversation about the role of cats in our yards and in our spaces.*”

Participants often weighed these roaming risks against the negative effects of keeping cats indoor-only. US5, who had previously worked in animal rescue, expanded: “*I don’t think it’s necessarily more responsible to the cat to just keep them inside all the time because … we get a lot of miserable cats that way, like, I’ve met a lot of them.*” CAN3 similarly believed cats “*should* [get to] *be outside…if you don’t take them out, I think they could become kind of destructive…and upset and it could affect their personality* [or cause] *depression.*”

Here, participants’ concerns about cat safety and frustration reflect previously reported risks of roaming and indoor-only management, respectively (Kasbaoui 2016; Tan *et al.* 2020, 2020). In weighing up these risks, participants looked to walking as a solution that resolved both, echoing suggestions from Seksel (2009; p 78) that walking may meet “*the needs of indoor cats*”. Walking was communicated as a “*responsible*” [AUS1] and “*safe solution*” [US1] that allowed cats to get outdoor stimulation in a controlled, supervised manner: “*I love the balance that it gives me, gives us…to be able to go outside in a way that’s safe for both of us*” [US5]. It was additionally noted that walking may be more enriching than roaming due to the variety of environments:

“*Outdoor cats, they just go around their neighbourhood, which is like a street or whatever, and that’s quite boring. But mine get to go up mountains…all round Scotland and Wales, I think that’s quite good for them and it’s more exciting*” [SCO2].

### Walking is positive for the cats

Many participants described their cats as “*high energy*” [CAN4] and therefore difficult to manage. ENG5 explained that without stimulation, his cat ‘Leia’ is “*a real minx … she’s crazy as anything…she’s quite a terror, breaks things and stuff.*” ENG1 also struggled when she first brought home her cat, ‘Woody’:

“*I would like, spend the whole evening playing with him…I just couldn’t burn his energy…a lot of people say that Ragdolls are lazy. He was more like a, I guess like a Bengal in terms of his energy. He was just always running around and just always wanting attention.*”

Owners of these high-energy cats commonly communicated that the cats started doing “*better*” [CAN4] after introducing walking, which reduced the strain on them as owners: “*it…took a lot of pressure off me to entertain them in the house. So, if I take him for a fifteen-minute walk outside, I don’t have to play with him as much*…[walking is] *the stimulation*” [AUS1]. This reflects literature highlighting exercise and enrichment as beneficial to the welfare of cats, especially in reducing energy and unwanted behaviours that may come from lack of stimulation (Ellis 2009; Houser & Vitale 2022). From the perspective of owners in this study, these findings thus suggest that walking cats may be considered an additional enrichment tool for cats who enjoy it.

Specific changes after walking included high-energy cats being noted as “*a lot calmer*” [ENG1], sleeping more, and displaying reduced vocalisation and behaviours indicative of frustration, e.g. “*he doesn’t scream and cry all night…His 3 am zoomies are reduced*” [SCO1]. Similarly, Lim and Rhodes (2016) found that owners of high-energy-level dogs walked them more vigorously and frequently than those owning low-energy dogs and that their dogs’ energy-demand characteristics specifically motivated them to do so. These findings suggest that for cats kept similarly to many pet dogs (without free roaming access to the outdoors), energy demands may be a contributing factor to decisions to walk, and perceptions of benefits gained.

Cats were additionally described as “*eager, they like to spend time outdoors*” [US3] and were reported to enjoy walks based on the behaviour they exhibited:

“*I would say for him, it’s really enriching. He gets to explore more, see nature… he’s really curious. So, you can see him wanting to just see what’s out there…seeing him run through the fields or jump and chase butterflies is like, it’s pure happiness. You can absolutely see that…his tail goes up, he’s just such a happy cat*” [ENG2].

Walking was also seen to facilitate the expression of natural behaviours, including hunting, climbing and marking. Hunting behaviours were frequently described as a “*big activity*” [US3] when walking: “*she thinks I’m taking her out for a hunt. There are bugs to pounce on, we have invasive lizards that live under the siding of the house*…[she’s] *constantly hunting things*” [CAN1]. US7 noted how walking transformed the personality of her timid cat, ‘Clyde’, by enabling natural explorative behaviours:

“*I nicknamed him the ‘Leopard King’ because he would totally turn into this leopard and act like he was just king of the jungle…it was like this little shy kitten, and then suddenly he was in his element. You could see he would just change. I mean, it was like his genes were just kicking in and he wanted to climb everything and conquer.*”

Although multiple organisations express concern over leashed walking being frustrating for cats (Battersea Dogs and Cats Home 2023; Blue Cross 2023; Cats Protection 2024), others within the field have suggested the possibility of its benefit (Seksel 2009; Tan *et al.* 2020). This subtheme supports that, at least as perceived by participants in this study, natural behaviour may still be performed when cats are leashed, providing enrichment and safety concurrently.

### Walking is positive for owners

Participants additionally highlighted that they derived several benefits from walking. Many mentioned that walking being good for their cat positively impacted their own emotional state: “*I enjoy…that she gets enjoyment out of it*” [US6] and “*it’s like when they have the best day, you have the best day*” [US1]. This finding reflects that of Westgarth *et al.* (2017) on dog walking, where motivations for walking include happiness, which in turn is dependent on the perception that dogs enjoy the process.

Walking itself was also described as a facilitator of the owner-pet bond. CAN4 described that on walks “*a lot of the time it’s just a nice bonding, head butting experience and he’s doing dumb cat stuff*” [CAN4]. Sharing experiences together enabled “*a closer bond, as pet and owner, that’s kind of different* [to the bond with their other pets]” [CAN1]. For some, having a cat used to walking and new environments enabled them to more easily travel with their cat, which in turn meant never having to leave them behind. For example, ENG2 explained that “*the holidays got so much better. So, whenever we go to Europe, he just goes with us, and it’s absolutely brilliant…to be away from home and to know that he’s with us, it’s just so much better.*”

Some also described experiencing a meditative, present headspace that was shared with their cat whilst walking: “*I’m into this magical world where we’re just tuned in, both of us, tuned into nature. And I just love that*” [US7]. It also helped owners appreciate “*the small things*” [ENG1] in their outdoor environment or from “*a different perspective*” [US3] as their cats did:

“*I’d say what I most enjoy are the things that my cat shows me that I wouldn’t have noticed. Like the other day, she stopped to just look at something, and there was a snake. I would never have seen the snake, so that was kind of neat*” [CAN1].

This subtheme highlights the similarities between perceived walking benefits for this group of cat walkers and those reported by dog walkers, especially regarding shared happiness and positive experiences (Westgarth *et al.* 2017). Additionally, what also becomes apparent is how entangled positive effects for participants are with those of their cats when walking, and how walks enable shared experiences. This bidirectional relationship of affect and the attunement with cats communicated by participants reflects sentiments shared by those in Meehan *et al*.’s (2017) study on pets and social support, wherein pets were viewed as “*distinctive sources of social support, at similar levels to their significant others, family, and friends*” (2017; p 1). This suggests the owner-perceived relationship with their cat to be impactful for walking, as well as general cat ownership.

### Theme 2: Challenges around walking

#### Dogs and their owners

Off-leash or uncontrolled dogs were a challenge for participants: “*I have found not much* [hard] *except* [the] *difficulty is always dogs, unleashed, but it should be on-leash…you have to always be careful for that*” [NET1]. For all participants, the main concern with these dogs was the potential to harm the cat. CAN1 explained that her “*biggest fear is an off-leash dog coming and biting her and that’s the end of* [her cat].” This threat caused all participants to be hyperaware, with their “*eyes open for dogs constantly*” [CAN2]. For some, this limited the scope of their walking due to being “*afraid of other people’s dogs. And like, how they take care or how they* *air quotes* *‘take care’ of their dogs on a trail*” [US5]. Several highlighted dog owners as enablers of this potential risk, labelling them as “*irresponsible*” [CAN1] or as flouting the rules: “*where we go, it does say your dog should be on the lead and people don’t…some of them…actually don’t even have a lead on them to put on their dog*” [ENG5].

To evidence their concerns, participants described negative “*instances*” [CAN4] with dogs whilst walking, ranging from being “*rush*[ed] *at*” [US1] by on-leash dogs or close calls of off-leash dogs “*charging*” [SCO1], up to more extreme experiences of dog attacks, as SCO2 described:

“*We were attacked…it felt traumatic…we were like chilling on the beach, they were in their backpack. There were no trees. And the dogs managed to rip open the backpack…I couldn’t get them to a tree because there was none…I didn’t have dog spray at that point…I had to go to the hospital, and I had three weeks of antibiotics. I had to have an X-ray cause the dog bit my fingers really bad…a tetanus injection as well. It was just a lot of things that I had to do because of some irresponsible owners.*”

To mitigate dog encounter risks, participants used backpacks or awareness of high trees as escape points, in addition to choosing walking routes where dogs were uncommon or not permitted. However, even in these circumstances, owners maintained the need for constant vigilance. Here, the owners in this study echoed the risks of dogs to free-roaming cats outlined by other authors (Rochlitz 2009; Tan *et al.* 2020; 2021). This theme suggests that walking cats on a lead does not entirely circumvent these risks, and indeed, dogs appear to be a substantial barrier to cat-walking owners.

#### Social difference and judgement

Cat walking was described as uncommon by all participants, regardless of whether indoor-only or indoor-outdoor management was more popular in their location, highlighting the practice as something that marked them as different from their community. Owners acknowledged that walking their cat(s) was “*not a norm*” [ENG1], and strangers were often described as “*surprised, one hundred percent*” [FRA1] or confused upon seeing their cat being walked. Participants also described facing judgment from strangers when walking their cat, which was “*very strange*” [AUS1] to do, especially in comparison to dog walking: “*some people said, ‘well, why don’t you get a dog’? They felt it was strange to do that with the cats*” [NET1]. Visual judgement, such as staring and negative comments, was commonly described. For some, this negative judgment escalated to physical threats: US1 revealed that she “*had people who didn’t like ‘Morrigan’, who would take a kick at her*” [US1].

Participants theorised that the judgement came from a lack of understanding regarding walking cats, as “*that concept alone is madness for people*” [ENG4]. Hill’s (2024) investigation into the indoor/outdoor cat management debate similarly highlights themes of the presence and difficulty of social judgment, such that participants conveyed either feeling judged or expressing a judgmental attitude toward others around cat outdoor access. Here, our participants experienced similar difficulties with judgment around walking, suggesting that social attitudes concerning cat management pervade conversations about not only whether cats are allowed outdoors, but also the quality of that outdoor access.

In contrast, participants frequently used their “*supportive and lovely*” [ENG5] online communities of cat walkers, where understanding of the practice was shared. Describing the community, ENG2 explained, “*as you could probably see through the recruitment, the cat community … it’s a small community, but it’s so strong as well.*” Echoed by others, the cat-walking groups were commonly described as “*little*” [CAN4] but “*so supportive and so friendly and not negative at all*” [CAN3]. Participants’ descriptions of their walking communities suggest these to be a critical source of social support and a potential buffer against negative emotional effects of social judgement (Cohen & McKay 2020).

### Theme 3: Safety for walking

#### Preparation

Cat safety was a priority for participants, for which preparation was a key factor. This began with managing walking expectations, including “*to not expect it to be like walking a dog*” (ENG5), or for the process to be quick. Highlighted in discussions around preparation was the usefulness of cat-walking resources, such as the website KittyCatGO (2024):

“*It’s an American company…they do adventure challenges. That was a big thing that got me and ‘Batman’ more confident with being outside together was these adventure challenges, and you can be anywhere in the world to do it. It’s amazing*” [SCO1].

Additionally, some participants prepared for walking by “*talking to professionals*” [AUS1] such as behaviourists or veterinarians, or by researching cat behaviour:

“*I called, probably, five or six different vets…probably about twenty to thirty hours of research and articles, like, science journals. Like, all of it. Rabbit hole at 2 am. Yeah, I spend a lot of time checking all of those things out*” [AUS1].

Relying on “*other people’s experience*” [AUS1] and knowledge generated within the cat-walking community was also emphasised as important to preparation: “*I did reach out to a couple of people who were walking their cats like on Instagram, who are posting about it and I just reached out to them, just asking for some tips*” [ENG1]. CAN2 explained the benefit of this approach: “*It’s good to see reviews of products, for example, people discuss harnesses to use, what backpacks to use, you know, clicker training…it’s been helpful.*” This theme highlights how active this participant group was in searching for resources and information on cat walking, and that scientific literature in the area would be valuable to potential and active cat walkers.

#### Suitable equipment

The equipment used was a key consideration for participants when walking. A well-fitting harness and a leash were important: “[get] *a harness that’s safe for the cat. That doesn’t restrict them from movement and keeps them protected…so that they can’t get loose*” [US7]. Participants’ choices were varied and dependent upon what they felt was suitable for their particular cat: “*There are folks who side with the H type harness or the Figure 8 harness and ‘this is obviously the best,’ but I think it’s unique and it’s individual*” [US3] and “*it depends on where we’re going. You know, if it’s somewhere like a park with, you know, people and stuff, I don’t wanna do a flexi* [lead]*, but if it’s a hike, then flexi*” [US6]. Backpacks were also often used as a portable safe space for the cat, with strollers less so. Backpacks were particularly valued among those who used them:

“*My advice is to use the backpack. It’s very important to always bring it with you, even if it’s only for a small walk, but you never know…what’s gonna happen, what kind of situation you come into. Yeah. It really helps to, to have it with you*” [NET1].

Using a harness and leash to walk a cat is suggested in the minimal literature available that features walking (Seksel 2009; Tan *et al.* 2020), and backpacks are mentioned by the BC SPCA (2024) in their guidance on walking. In contrast, while there is some work on the impacts of various dog harnesses (Grainger *et al.* 2016; Lafuente *et al.* 2019; Shih *et al.* 2021), and although some organisations have voiced concerns over the safety of some cat equipment, such as ‘bubble’ backpacks (SPCA New Zealand 2024), to our knowledge there has not been any comparable investigation for cats, which this theme suggests would be valuable.

#### Training

Training cats to prepare them for the walking process was common among participants. Obedience commands, such as sitting or recall, were often taught prior to starting to walk as “*a big safety feature*” [US3]. Recall was commonly utilised for cats who were let off-leash when walking, or to redirect them without pressure on the leash. FRA1 explained: “*we taught him to come, the ‘come here’ command, because I thought it was… really important…for him to be able to do that*” [FRA1].

Habituation to harnesses and backpacks through positive reinforcement was also described, for example, through trying to “*associate the harness with good things like giving him some food*” (FRA1), and with an understanding of the process being gradual:

“*We just started putting it* [on], *at home for five minutes…then extend it literally by minute and minute…every single day. Then try to play with him. And after maybe a couple of weeks…he was chasing toys at home* [in the harness]” [ENG2].

Here, participants demonstrated awareness and application of behaviour shaping training techniques and habituation in achieving their desired training goals. US6 described how she would “*sit with the door open and I let them choose when they want to adventure out of the door*,” centring the cats’ autonomy in the activity. As SCO1 explained, “*everything is literally step-by-step. And I think that’s important for any animal.*” For participants with large walking scopes, these generally also increased over time, as described by US3: “*we started out small adventures, very close to home…within six months started going to the local park, going walking around before we went out to bigger places*” [US3]. Some participants shared that they incorporated clicker training into their preparation, for example:

“*I would like throw treats in. I use a clicker as well. Yeah. They kind of like jump in. I teach them to like with the click, I would teach them to jump in themselves and, like, carry them around the, the flat. And carry them on the stairs, et cetera*” [ENG1].

Participants widely reported that their use of positive reinforcement techniques was effective in achieving their walk training goals. Utilising training in this way for restrictive equipment reflects recommendations from researchers (Seksel 2009) and some animal welfare organisations (BC SPCA 2024; SF SPCA 2024) around harnesses and leashes. More broadly, these findings add to the body of evidence that employing shaping as a training tool (Pryor 2019), in addition to clicker training (Kogan *et al.* 2017) and positive reinforcement (Heidenreich 2007; Deldalle & Gaunet 2014; Willson *et al.* 2017), supports owner efforts to train animals to engage in desired behaviours in ways that can promote animal welfare (e.g. carrier training reduces behavioural and physiological distress in cats being transported for vet visits; Pratsch *et al.* 2018). It is also worth noting that, contrary to common misconception, the findings in this study support the growing literature detailing cats as a trainable species, for whom doing so can have enrichment benefits (Kogan *et al.* 2017; Willson *et al.* 2017). In the case of participants in the current study, this trainability was harnessed to make walking easier and safer.

### Theme 4: Cat individuality and walking

#### Suitability and subjectivity

Many participants were careful to note that walking may not be suitable for all cats: “*it’s not for every cat. I’m sure there are some that just wouldn’t enjoy it. And that’s totally fine as well*…” [ENG2]. For several participants, this meant they did not walk all their cats: “*it has everything to do with cats’ personality, like three of mine would never. But she loves it*” [US1]. This attitude aligns with that of a case-by-case stance on walking recommendations (SPCA New Zealand 2024).

The variation in walking scope between participants highlights the prioritisation of their cat’s needs when walking, with participants commonly altering their style and location of walking to ensure appropriateness: “*I’ve got two cats that have two different reactions to the outdoors, and they both go outside. Different times, different places, different situations*” [SCO1]. In some cases, walks were confined to the property: “*I have two cats. I have one that I take for walks off property and one just in the yard…the one that I don’t take off property is quite shy, so I think it would be a disaster*” [CAN1]. These on-property walkers emphasised how their identification with being ‘cat walkers’ made it a subjective term, which they fit to their cat, rather than needing to conform to a set idea: “*walking is a really loose term…I was saying to someone that it’s really like, I feel like we’re more hanging out…We’re more hanging out together with him leashed*” [CAN3]. Of interest within this subtheme is how subjective the nature of ‘cat walking’ appears to be, and further investigations in this topic area should be mindful of the many things owners may mean when saying they ‘walk their cat’.

For others who left their properties, walking spots often included parks and nature reserves. For CAN2, these were easily accessible: “*we’re really lucky we live next door to a very large natural park. So, we just have to put the leash on and there it is*…[we] *haven’t taken them anywhere else, haven’t really had to bother.*” Others took advantage of local towns and residential areas; ENG3 highlighted that his cat “*likes humans’ places more than, you know, the nature.*”

Around half of participants ranged farther afield, or ‘adventured,’ with their cats. For some, such as US1’s “*city girl*” cat, this included walking in highly populated urban areas and often into shops or malls. Others adventured rurally, walking or hiking in national parks, doing activities such as kayaking or camping, or going on trips together, cross-country or internationally. SCO2 explained that as her cats are used to a large scope of walking, she could take them “*anywhere, really, wherever I want to go.*” For SCO1, adventuring extended to the UK’s highest peaks: “*We hiked Ben Nevis a week and a half ago…he’s a small cat and he’d walked, by then, six hours*” (SCO1). These adventure cats were also often described as being used to various modes of transport, including owners’ cars, vans or bikes, in addition to public transport like trains and trams (e.g. “*last spring we travelled from the UK on to, to Poland…literally eight hours on a train…he’s like, totally fine*” [ENG2]). It is worth noting that while the cats of these participants were reported to be able to cope on transport, distress due to transportation is significant for many cats (Pratsch *et al.* 2018). To our knowledge, there is no scientific evidence to suggest that transport stress is not experienced by ‘adventure cats’ or any cat made to travel frequently, and as such, research on the welfare impacts of travelling with cats in this way requires attention.

#### Prioritising cat desires

Participants were commonly driven by a desire to “*work out* [the cat’s] *likes and dislikes*” [ENG3] and “*follow their needs*” (NET1) when walking. Participants were knowledgeable about what behaviours their cats engaged in on walks and what they seemed to enjoy most. Common activities included lying down and “*hanging out*” outside, “*investigation, sniffing, hunting*” [CAN3], running and “*zoomies*” [ENG4], and “*tree climbing*” [NET1]. They also demonstrated awareness of the degree of individual variation in their cats, as illustrated by CAN2:

“*We’ve actually classified our cats according to three different routines they have…‘Mustard’ has the nickname of Constable ‘Mustard’. Because he goes out to be on patrol…‘Plum’ is a tourist…watches the scenery for the most part. And [Scarlett] is the hunter…for her, the walks are hunting*” [CAN2].

This mindset of prioritising the cats’ desires led many owners to practice cat-directed walking, in which the owner allowed the cat to make “*design choices*” [US3] and take specific paths or directions they were interested in: “*if we have like a fork in the trail and there’s multiple ways we can go, I’ll let her choose … It is a cat-directed walk*” [CAN1]. This style of walking is somewhat reflective of shaping and other behaviour-training techniques in which the animal holds more autonomy for the actions they perform, enabling positive welfare (Pryor 2019). Being acutely aware of the individual cats’ needs in this way is suggested to be an important factor in ensuring that walking is positive and not a negative stressor for each animal. This subtheme shows similarity to how dog walkers studied by Westgarth *et al.* (2017) used interpretations of their animal’s behaviour (whether they were enjoying walking or not) to inform their walking practices and changes they made based on the dog’s own desires and needs.

In contrast, Hill (2024) found that owners weighed their cats’ desires less than their own when making decisions for their cat’s management around roaming. However, for our participants, the majority of their walking activity appeared to be based on their cats’ needs. It should be noted, however, that risks to cats freely roaming vs on walks with their owners remain underexplored but likely differ greatly. The use of restraining equipment or the behaviour of specific cats (i.e. those that follow their owner closely) may mitigate the perception or reality of some risks of being outdoors for owners who walk their cats.

### Theme 5: Attitudes about walking across geographic contexts

Participants’ views and those of their communities were affected by the location in which they were based, according to the prevailing cultural stances in that location regarding the cat outdoor access debate. For example, for participants based in the UK, keeping cats indoors and walking them was viewed as societally “*controversial*” (SCO2), as free-roaming was more generally culturally accepted:

“*I’ve had some negative comments*…[someone] *walked past and he was, like, quite cocky…he said, ‘are you walking cats?’ I said, ‘yeah’. He said, ‘well, that ain’t normal, what you doing that for? They walk themselves’*” [ENG4].

Indeed, both UK and some European participants expressed that cat walking was received negatively due to the idea that cats can walk themselves without the need for supervision. There is an absence of laws relating directly to the containment of pet cats throughout the UK and Europe, and acceptance of cats roaming is reflected in social attitudes across the geographical region (Foreman-Worsley *et al.* 2021). Roaming being both ‘the norm’ and commonly practised was noted to negatively affect how the walking of cats was received by Europeans within this participant group.

In contrast, US1, who had lived in both the Republic of Ireland and the US, noted that in the US “[walking is] *not that odd, and I think it’s because we don’t really have, as often, free-range cats. That’s considered a bit more of a negative here.*” CAN2 expressed a similar sentiment, adding that in Canada, “*people don’t like roaming cats, so they’re happy to see cats on the leash because the cats aren’t roaming*” [CAN2]. The degree to which cats are legally expected to be contained or allowed to roam free varies across North America, with bylaws requiring cats to be leashed or kept contained in some municipalities in Canada (e.g. Victoria Animal Control Services Ltd 2024) and the USA (e.g. City of Aurora 2022; City of Baton Rouge and the Parish of East Baton Rouge 2025). However, this sense of communal support for walking was not experienced by all US participants, as some found having a cat outdoors at all was criticised as unnecessarily risky: “[people] *just fall into that perception where ‘cats are indoors. You don’t take them outside,’ you know. I mean, everybody, sort of, just believes that*” [US7]. This attitude is also seen reinforced by the ASPCA (undated), who ask owners to “please keep your cat indoors”. These geographical differences in the perception of cat walking are reflective of contrasting outdoor access attitudes (Forman-Worsley *et al.* 2021), although their regional variation is noted as being complex.

The geographical differences in how cat walking is received also reflect the varying stances of animal welfare organisations. For example, our UK and European participants noted walking to often be viewed negatively. In the UK, the charities Battersea Dogs and Cats Home (2023), Blue Cross (2023) and Cats Protection (2024) do not recommend the practice, as cats “*will usually find being walked on a lead very stressful*” (Cats Protection 2024), or they warn that if a cat gets loose, “*it is unlikely they will return to you*” (Blue Cross 2023). Walking was received more positively by participants in North America, where organisations appear more supportive of walking. For example, the San Francisco SPCA (2024) claims that “*teaching your cat to walk…is a great way to let your cat safely enjoy the outdoors*,” while The Humane Society of the United States (2024) approves of walking to promote mental well-being in indoor cats for owners who “*live in a peaceful neighbourhood in which you can walk without encountering loose dogs*”. In Canada, the BC SPCA (2024) claims that “*one way to keep* [cats going outside] *safe is to teach them to walk on a leash.*” These similarities between public and organisational opinion on cat walking when investigating geographical impacts on the practice are interesting, and suggest complex relationships between the domains of law, veterinary and animal welfare advice, and public opinion.

### Study considerations

This work provides an initial understanding of what cat walking can be and highlights the key benefits, challenges, priorities and geographical attitudes of the participant pool. These findings highlight key areas of interest for future investigation to better understand how the practice of walking cats can impact their welfare. We acknowledge that the sample of this study may be considered limited due to the use of cat-specific groups to recruit participants rather than being representative of an overall cat-owning population that has attempted walking, and thus, results likely represent a specific subset of successful cat walkers. However, we note that generalisability is not an aim of the types of qualitative paradigms and methods employed in the present study (Carminati 2018), where interest lies instead in a rich exploration of current walkers’ experience (Willig 2022). The gender disparity and Westernised locations of participants are also acknowledged, lending further caution to attempts to generalise these findings. We encourage further research with larger participant groups, of broader geographic scope, to better characterise the demographic range of cat walkers on a global scale.

### Animal welfare implications

This study explored cat walking with owners who currently practise it. Our findings provide an overview of perceptions and priorities around cat walking amongst a small population of cat owners based predominantly in North America and Europe. Participants widely found their cat-walking endeavours to be beneficial for themselves and their cats, with increased cat safety compared to roaming and improved cat autonomy and engagement commonly valued. Challenges to walking focused mainly on the risks of dogs and on social judgment, but owners found ways to manage these challenges, which motivated them to continue the practice. They additionally highly prioritised the needs and desires of their cats throughout walks, highlighting care for both their cats’ psychological and physical welfare. The study also highlights the subjective nature of cat walking as an activity, as well as in its conceptualisation. It is suggested that further research on the topic either honour and investigate this variety, or utilise clear definitions of walking scope, especially when gathering participants to ensure clarity in nomenclature. As the topic remains unexplored, further investigations of both a qualitative and quantitative nature into the practice of cat walking is suggested as highly valuable to current and potential cat walkers, as well as to academics and veterinarians.

### Future research directions

Ultimately, this study highlights a critical need to develop research to identify the welfare impacts of walking equipment, travelling with cats, cat temperament and suitability for walking, and the risk of dogs to cats on leashes. Whilst owners’ views and perceptions are valuable in this regard, direct study of cats in these scenarios is also necessary to understand the welfare implications of walking cats. The attachment of owners to their cats and the self-described strengthening of their bond is an additional factor worthy of future investigation. The differences in owner-cat bond between those who walk cats and those who do not may be of particular interest. Finally, cat outdoor access in general is seen to garner little agreement across geographical locations, and this points to bidirectional impacts on local social, legal and (animal) professional levels. Given the small but committed group of owners who are interested in using this practice to improve the welfare of their cats, urgent scientific attention is needed to clarify the extent to which, and in which contexts, walking may diminish or improve cat welfare. It is suggested that future investigations into cat walking take into account participants’ varying contexts and utilise a range of welfare science and social science tools to best understand and create guidance around the practice.

## Supporting information

Elford et al. supplementary material Elford et al. supplementary material

## Supplementary material

The supplementary material for this article can be found at http://doi.org/10.1017/awf.2025.10030.
