# Supplementary material for: “I want them to live their best lives:” A qualitative exploration of owner experiences with walking their cats
Source: Anim Welf. 2025 Aug 8;34:e56. doi: 10.1017/awf.2025.10030 (PMC12451394; doi:10.1017/awf.2025.10030)
Supplement: Elford et al. supplementary material [file S0962728625100304sup001.pdf]

“I want them to live their best lives:” A qualitative exploration of owner experiences with walking their cats

Alex Elford[1,2] https://orcid.org/0009-0004-1833-9704, Andrew S Cooke[1] https://orcid.org/0000-0003-2225-1890, and Beth Ventura[1,3] https://orcid.org/0000-0001-9476-6901

1. Department of Life Sciences, University of Lincoln, Joseph Banks Building, Green Lane, Lincoln LN6 7TS, UK

2. Department of Livestock and One Health, Institute of Infection, Veterinary and Ecological Sciences, University of Liverpool, Leahurst Campus, Neston CH64 7TE, UK

3. Department of Large Animal Clinical Sciences, College of Veterinary Medicine, Michigan State University, East Lansing, MI, USA 48824

Author for correspondence, Beth Ventura: email: venturab@msu.edu

Supplementary material

Initial Survey Questions:

Exploring Cat Walking Practices and Owner Perceptions

Owner

1. Age: (State)

2. Gender: (State)

3. Location: (State/Province/County and Country)

4. What type of property do you live in? Select one:

- ❑ House (Detached)
- ❑ Semi-detached house (Duplex)
- ❑ Terraced house (Town home)
- ❑ Flat (Apartment)
- ❑ Vehicle Home (Caravan, etc.)
- ❑ Other (Please state) ....

Cat

5. Age: (State)

6. Sex and Neuter Status - Select one: Male Neutered / Male Entire/ Female Neutered / Female Entire

7. What outdoor spaces does your cat have access to at home? Select all that apply:

- ❑ None
- ❑ Porch or balcony
- ❑ Catio/Other enclosed outdoor area
- ❑ Garden (not enclosed)
- ❑ Own wider property (owned fields/land)
- ❑ Unowned wider property (public or neighbour-owned gardens/fields/land)

8. What degree of access does your cat have to outdoor spaces at home? Select one:

- ❑ None
- ❑ Restricted (I will let them out if they ask and I think it is okay, and I have to give them access)
- ❑ Semi-restricted (I will let them out whenever they ask, but I have to give them access)
- ❑ Unrestricted (They can go outdoors whenever they want to, through a cat flap, constantly open window, etc.)

Initial Interview Schedule/Questions:

Exploring Cat Walking Practices and Owner Perceptions

- *Introduce yourself to participant, thank them for their interest*
- *Briefly remind them of the project direction*
- *Confirm they have read PIS, signed consent form and completed demographics survey*
- *Ask if they have any questions*

Main Interview

Q1 - Tell me about your cat/s

-How would you describe the relationship between you and your cat?

-Where did you obtain your cat? How long have you had them?

Q2 - Why do you walk your cat?

Q3 - Tell me about how you introduced your cat/s to walks?

-How old was the cat when you started?

-What equipment did you use?

-What training, if any, did you use?

Q4 - What normally happens on a walk?

-Where do you go?

-How long do walks tend to be?

-Do you tend to stick to any routine or is there a lot of variation?

-What is the main purpose of your walks (for example, exploration, exercise or bonding)?

Q5 - What does your cat normally do on a walk?

-Is that what you would like the cat to do?

Q6 – What is your overall experience of walking your cat like?

-What, if anything, do you enjoy?

-What, if anything, do you not enjoy?

Q7 – What did you think about walking cats before you started?

-How did you become aware of it?

-Were there any physical/mental/social barriers or challenges you were concerned about?

Q8 – How do other people react to you walking your cat?

-How do strangers when you come across them when out walking?

-How do people react in conversation?

-Have you experienced reactions being different in different areas at all?

-Has anything bad happened that you are willing to share?

Q9 – Is there anything else you would like to tell me about your experience? (RQ1)

Thank the participant for taking part, remind them they can ask for the findings once completed
